# Supplementary material for: Induction of Proteasome Subunit Low Molecular Weight Protein (LMP)-2 Is Required to Induce Active Remodeling in Adult Rat Ventricular Cardiomyocytes
Source: Med Sci (Basel). 2020 May 1;8(2):21. doi: 10.3390/medsci8020021 (PMC7353499; doi:10.3390/medsci8020021)
Supplement: Supplementary file 1 [file medsci-08-00021-s001.zip › medsci-784079-supplementary-1.pdf]

medical sciences

MDPI

Supplementary Material

Table 1. List of Primers used in this study.

	Forward	Reverse
ANP	ATG GGC TCC TTC TCC ATC AC	TCT TCG GTA CCG GAA GCT G
Troponin T (TnT)	AGG AGA ACA GGA GGA AGG CT	CTG GAT GTA CCC TCC AAA ATG C
αMHC	CAC CCT GGA GGA CCA GAT TA	TGG ATC CTG ATG AAC TTC CC
βMHC	GAA GGA GGA CCA GGT GAT G	AGG TGT AGA TCA TCC AGG AAG
βActin	GAA GTG TGA CGT	TGA CAT CCGTGC TGA TCC ACA TCT GCT GGA
LMP-2	CTT TGC AAT CGG TGG TTC CG	CGG TTC ATG GCC AGA GTG AT
SDF-1α	CCA AGG TCG TCG CCG TGC TG	GGC TCT GGC GAC ATG GCT CT
CXCR4	GCC ATG GCT GAC TGG TAC TT	CAC CCA CAT AGA CGG CCT TT
Bax	ACT AAA GTG CCC GAG CTG ATC	CAC TGT CTG CCA TGT GGG G
B2M	GCC GTC GTG CTT GCC ATT C	CTG AGG TGG GTG GAA CTG AGA C

Table 2. List of primary antibodies used in this study (all: mouse monoclonal IgG).

LMP-2	LMP2 (G-3) sc-373996	Santa Cruz
GAPDH	Anti-GAPDH Mouse mAb (&C5)	Calbiochem
MHC	Heavy Chain Cardiac Myosin (ab15)	abcam
Actin	MaB to cardiac Actin (Ac1-20.4.2)	ProGen

Video: The video shows the time-dependent cell rounding of initial rod-shaped cells.
